# Improving Nutrition and Health through Non-timber Forest Products in Ghana

**DOI:** 10.3329/jhpn.v29i2.7856

**Published:** 2011-04

**Authors:** Albert Ahenkan, Emmanuel Boon

**Affiliations:** **Human Ecology Department, Vrije Universiteit Brussel, Laarbeeklaan 103, B1090, Jette, Brussels, Belgium**

**Keywords:** Forest, Food security, Health, Nutrition disorders, Micronutrients, Non-timber forest products, Nutrition, Plant medicine, Poverty alleviation, Ghana

## Abstract

Nutrition and health are fundamental pillars of human development across the entire life-span. The potential role of non-timber forest products (NTFPs) in improving nutrition and health and reduction of poverty has been recognized in recent years. NTFPs continue to be an important source of household food security, nutrition, and health. Despite their significant contribution to food security, nutrition, and sustainable livelihoods, these tend to be overlooked by policy-makers. NTFPs have not been accorded adequate attention in development planning and in nutrition-improvement programmes in Ghana. Using exploratory and participatory research methods, this study identified the potentials of NTFPs in improving nutrition and food security in the country. Data collected from the survey were analyzed using the SPSS software (version 16.0). Pearson's correlation (p<0.05) showed that a significant association exists between NTFPs and household food security, nutrition, and income among the populations of Bibiani-Bekwai and Sefwi Wiawso districts in the western region of Ghana. NTFPs contributed significantly to nutrition and health of the poor in the two districts, especially during the lean seasons. The results of the survey also indicated that 90% of the sampled population used plant medicine to cure various ailments, including malaria, typhoid, fever, diarrhoea, arthritis, rheumatism, and snake-bite. However, a number of factors, including policy vacuum, increased overharvesting of NTFPs, destruction of natural habitats, bushfires, poor farming practices, population growth, and market demand, are hindering the use and development of NTFPs in Ghana. The study also provides relevant information that policy-makers and development actors require for improving nutrition and health in Ghana.

## INTRODUCTION

Globally, nutrition and health have improved in recent decades but malnutrition, including deficiencies in micronutrients, is still widespread, particularly in developing countries (1-2). Nutrition and health are fundamental pillars of human development across the entire life-span (2). In recent years, increased attention has been focused on the potential role of non-timber forest products (NTFPs) in reduction of poverty, in improving nutrition and health, and sustainable management of forest resources (3-7). The term NTFPs encompasses all biological materials other than timber which are extracted from forests, other wooded land, and trees outside forests and domesticated that include products used as food and food additives (edible nuts, mushrooms, grass-cutters, snails, fruits, herbs, spices and condiments, aromatic plants, game), fibres (used in construction, furniture, clothing, or utensils), resins, gums, and plant and animal products used for medicinal, cosmetic or cultural purpose for human use (8-10). NTFPs form an integral part of the livelihood strategy of rural communities in the tropics and continue to be an important component of household nutrition and health in Africa (11-12). They are particularly an important component of household subsistence, especially in terms of food consumption, nutrition, and health. In Ghana, a considerable amount of food and medicinal plants can be gathered from the forest and bushy fallow areas or are semi-domesticated (13-14). NTFPs contribute substantially to nutrition, either as part of the family diet or as a means to achieve household food security (13-14). They also improve health through the prevention and treatment of diseases (15-19).

Although Ghana, compared to other sub-Saharan countries, has a low prevalence of stunting on average (20), there has been no significant change in rates of mortality of children aged less than five years and infants of Ghana during 1993-2003. Poverty and decline in food availability and accessibility are the main underlying causes of malnutrition in Ghana (21). Asibey-Berko estimated that Ghana would lose 161 million dollar as the cost of health expenditure and low productivity if measures are not taken to improve malnutrition and other iron-deficiency anaemia of its population (22). About 54% of all deaths beyond early infancy are associated with protein-energy malnutrition, making it the single greatest cause of child mortality in Ghana (23). Interventions to prevent micronutrient deficiencies would best be addressed through food-based strategies, such as dietary diversification through home-gardens and NTFPs (24).

Despite this vital role of NTFPs, their potential contribution to food security, nutrition, health, and sustainable livelihoods tends to be overlooked by policy-makers in Ghana. NTFPs have not been accorded adequate attention in development planning and in nutrition-improvement programmes in the country. Research on NTFPs in Ghana is relatively new and has received very little formal investigation. Most recent nutritional studies focus on staple foods, such as cassava and maize. Very little research has been conducted to assess the potential contribution of NTFPs to nutrition, health, and food security of rural communities in Ghana. Inventories of NTFPs by the Forestry Commission of Ghana focused only on rattans, climbers, and other minor tree species (1). The Ghana Poverty Reduction Strategy Paper (GPRSP) also failed to consider NTFPs and their nutritional and health linkages. Various attempts to value NTFPs have examined only the current local market-value of these products and have not attempted any in-depth evaluation of the benefits to rural communities of health and nutrition strategies (1). This paper, therefore, seeks to examine the contribution of NTFPs to household nutrition, health, food security, and poverty reduction in Bibiani-Bekwai district (BBD) and Sefwi Wiawso district (SWD) and to identify the types and uses of NTFPs in the two districts.

## MATERIALS AND METHODS

### Study location

**Fig. 1.**
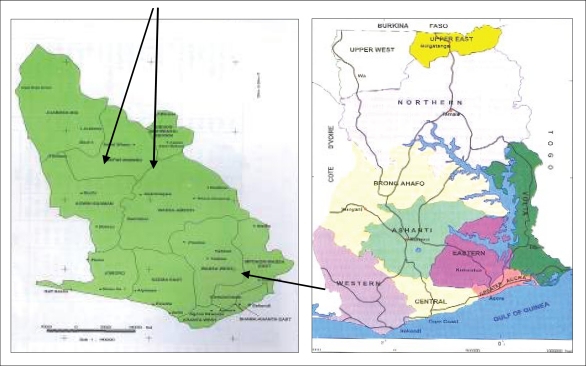
Bibiani-Bekwai and Sefwi Wiawso districts (25)

The study was carried out in the two districts as mentioned (Fig. 1), located in the western region of Ghana during April-July 2009. These two adjoining districts were selected because these constitute a major production area for NTFPs in Ghana. Moreover, these have common natural, social and economic characteristics that indicate high dependence on forest.

### Data-collection and analysis

In addition to an extensive literature review, both secondary and primary data were collected for the study. Secondary data, including documentation on food security, farming of NTFPs, medicinal plants, and their therapeutic uses, were collected from the District Directorate of Agriculture and Health Services Departments of the two districts, Ghana Food Research Institute, and published data from the Ministry of Health and World Health Organization. Five exploratory and participatory research methods, including key-informants interviews (26), administration of a questionnaire, observations of participants (27-28), focus-group discussions (FGDs) (29), and consultations with stakeholders were used for soliciting responses of communities to the farming, collection, and consumption of NTFPs and also to various facets of nutrition and food security-related issues.

A field survey in the two selected districts was conducted during April-July 2009. A purposive sampling technique, based on field expertise of the participants on NTFPs and nutrition-related issues in the districts, was used for selecting key-informants. To ensure a broad-based and objective data-collection, a carefully-designed questionnaire was administered to the key-informants knowledgeable in NTFPs and nutrition and health issues in the two districts. A structured questionnaire was the main research instrument used for collecting primary data from 200 randomly-selected heads of households from 10 communities in the two districts. Ten household heads were randomly selected and interviewed per community. The communities include Sui, Nkonya, Ahokwaa, Bosomoiso, Wiawso, Ahwiaa, Adupri, Bekwai, Atronsu, and Tanoso. Data were also collected on a quarterly basis from farmers and collectors of NTFPs through a survey that assembled information on the size of household, occupation, trends of production, sales, income, consumption, acquisition of assets, health, education of children, employment, and farming practices during 2008-2009. Health-related information included knowledge and use of medicinal plants, sources of household nutrition, and frequency of consumption of NTFPs.

Observation of the participants comprised visits to NTFP farms of the 20 respondents, market centres, and traders during April-July 2009, aiming at verifying and identifying various NTFPs that farmers collect, consume, or trade in. Further exploratory FGDs on farming of NTFPs, collection, use, marketing, management, and key challenges were conducted with women groups in five communities engaged in farming of NTFPs. To ensure the reliability and validity of data, it was triangulated with information collected from the households and interviews of key-informants. Collected data were quantified and entered into the SPSS software (version 16.0), and the results were analyzed. To ascertain the degree of association between the contribution of NTFPs to food security and the nutritional sources of the respondents, a Pearson's correlation was performed.

## RESULTS

About 80% of the labour force in the two districts was involved in agriculture, particularly cocoa, maize, oil-palm, cassava, cocoyam, and plantain production. However, agricultural productivity in these districts was quite low because of small farm holdings. Except cocoa, most farmers grow crops primarily for consumption in the home. Sources of food included market, farm produces, and collection of NTFPs.

The survey found that, in addition to the collection of a large variety of NTFPs from the wild, the respondents were mostly active in bee-keeping (44.4%), followed by grass-cutter production (39.8%) and snail rearing (29.3%) while 28.6% and 2.3% were engaged, respectively, in the production of mushrooms and medicinal plants to supplement their food, nutritional sources, and incomes. Of the 200 farmers sampled in Sefwi Wiawso and Bibiani-Bekwai districts, 80% and 85% respectively got some cash income from harvesting and farming of NTFPs, which contributed, on average, 35% of the total annual income. To determine the percentage of NTFP-based income and dependency on NTFPs, the respondents were classified into ‘rich’ and ‘poor’ income quintiles, based on the average annual incomes of the households surveyed and Ghana's Fourth Living Standard Survey (25). The survey revealed that about 32%, particularly the poor-income group, got more than half of their total income on NTFPs (Table 1).

**Table 1.** Annual income and share of NTFP-based income among respondents

Income quintile of respondents	%	Annual income (GH¢)	NTFP-based income as share of total income (%)
1 ‘rich’	5	5,000-6,000	25
2	41	3,000-4,900	28
3	23	2,000-3,000	45
4	16	1,500-2,000	51
5 ‘poor’	15	650-1,000	55

‘1’ represents the upper (high-income) quintile, and ‘5’ represents the lower (low-income) quintile US$ 1=1 GH¢.45

### Contribution of NTFPs to household nutrition and health

**Fig. 2.**
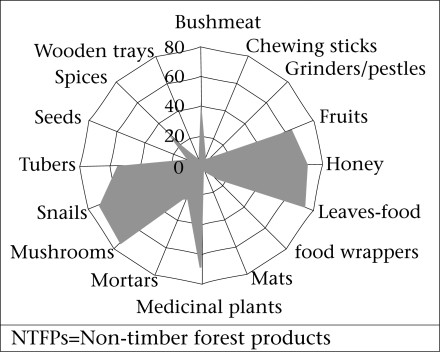
Importance of some NTFPs to respondents

During the interviews and FGDs in the field, farmers and women groups in the two districts indentified various NTFPs and ranked their importance in terms of their contribution to food, nutrition, health, and income. These products are mostly food products, medicinal plants, hygiene tools, household goods, and ornaments. Figure 2 depicts the comparative importance of NTFPs and the frequency of their usage among the respondents.

The NTFPs species found to be the most important and constituting part of the daily nutrition sources of the respondents were edible animals and plant products, followed by medicinal and ornamental plants as indicated by shaded portions in the figure. The more important is the product to the respondent, the longer the shaded portion of the figure compared to the centre point. These products contributed significantly to household food security, nutrition, health, and income in the two districts, especially during the lean season (June-August). These provide essential proteins, vitamins, minerals, and carbohydrates to the households (29). The respondents emphasized the importance of NTFPs, such as grass-cutters (*Thryonomys swinderianus)*, antelopes, monkeys, snail, mushrooms, and fruits in their diets. The most widely-consumed NTFPs were bushmeat, mushrooms, snails, leaves-food, honey, and fruits.

### Frequency of use of NTFPs

The contribution of NTFPs to the household dietary patterns did not vary significantly between the two districts. However, the contribution to household diet varied from one to another village and was largely a function of the environmental, sociocultural and economic contexts. For instance, the households in Ahokwa, Atronsu, Sui, and Nkonya tended to consume more NTFPs compared to those in other communities in the study area, which might probably be due to their proximity to forest reserves. The consumption of NTFPs among the poor households was also greater than the middleincome groups. The low-income households in the two districts consumed NTFPs for 5-6 days a week. The moderate-income groups consumed NTFPs, on average, three days a week. The most widely-consumed NTFPs, such as mushrooms, snails, and fruits, are available seasonally. However, because of domestication of these products, most farmers were able to consume the products during most parts of the year, especially grass-cutters, snails, mushrooms, and honey. Leaves were eaten regularly throughout the year by most (87%) households sampled. The surveyed NTFPs were consumed in varying quantities daily, weekly, occasionally, or rarely. Table 2 shows the frequency of consumption patterns when the product is available in the year.

**Table 2.** Frequency (%) of consumption* patterns when the product is available in the year

NTFP	Daily	Weekly	Occasionally†	Rarely§
Bushmeat	25	55	18	2
Honey	35	56	10	4
Leaves-food	50	30	15	5
Medicinal plants	31	25	39	5
Mushrooms	55	35	6	4
Snails	45	35	15	5
Fruits	54	20	25	1

*The frequency of consumption refers to consumption patterns when the product is available in the year; thus, when mushrooms are available (rainy season), 55% of those who eat these do so every day;

†2 weeks and more;

§Few times a year;

NTFPs=Non-timber forest products

These products also contributed significantly to household income and food security through their collection and sales at the local markets. Household food security does not only depend on the availability of an adequate and sustainable supply of food but also on the means employed by households to acquire the needed food. Using the Household Dietary Diversity Score, the large majority (75%) of the respondents were food-secure while 25% were food-insecure. The results showed that a significant association (p<0.05) existed between NTFPs and household food security and nutrition (Table 3).

**Table 3.** Pearson's correlation showing the degree of association between NTFPs and food security, and nutrition

Factor	NTFPs	Contribution to nutrition	Contribution to food security
NTFPs			
Pearson's correlation	1	1.000**	0.206*
Significance (2-tailed)		0.000	0.003
No.	200	200	200
Nutrition			
Pearson's correlation	1.000**	1	0.206*
Significance (2-tailed)	0.000		0.013
No.	200	200	200
Food security			
Pearson's correlation	0.206*	0.206*	1
Significance (2-tailed)	0.013	0.013	
No.	200	200	200

*Significant Correlation is at 0.05 level (2-tailed);

**Very strong correlation

### Contribution of NTFPs to health

Health is another important aspect of rural life. NTFPs make a crucial contribution to health through the supply of plant medicines. The majority (56%) of the population surveyed in the study area relied on plant medicines for their healthcare. Fifty of 65 plants species identified by the respondents all have medicinal properties to cure various ailments and diseases. Most species had more than a single therapeutic use. This was confirmed by the Ghana Centre for Scientific Research into Plant Medicine during consultations with stakeholders. The majority (67%) of the respondents had some knowledge/skill on how to prepare medicines from plant-parts. They were found not only to depend on the traditional healers for the remedies but they also prepared some medicine themselves. Ninety percent of the sampled population used these remedies to cure various ailments, including malaria, typhoid, fever, diarrhoea, arthritis, rheumatism, and snake-bite. About two hundred and fifty indigenous trees and plants with healing properties have been scientifically catalogued in Ghana by Abbiw *et al*. (32-33).

## DISCUSSION

### Contribution of NTFPs to nutrition, health, and poverty reduction

NTFPs-related practices are important elements of livelihood strategies among farmers in the study area. For the poor households, collection of NTFPs from the natural forest is a means of coping with food-supply, nutrition, medicine, and cash shortages. Households living near the forests are typically most dependent on these products and most active collectors of NTFPs in the study area. This high dependency on NTFPs, particularly by the poor-income group, is indicative of their importance in improvement of livelihoods, nutrition, and health. Interestingly, this pattern of NTFP-use and dependency reflects the findings of Falconer in south Ghana (31) and Anshu Singh *et al.* (35) in the Sundarbans. Consumption of NTFPs was not only high but also very common in the study area. Not only do NTFPs supplement vitamins, proteins, minerals, and the nutritional requirements of the communities, they also diversify their diets and enhance their seasonal food balance.

Unfortunately, the extraction of NTFPs from natural forests has limited potential for improving food security and nutrition of households. Since most of these products are picked freely from the forest during the season they blossom, this is obviously unsustainable. Consultations with the stakeholders and FGDs revealed that most communities in the two districts were losing access to these valued NTFPs either because of over-exploitation or destruction of habitats. Three main strategies have been employed to militate against shortfalls in supply in most communities in the study area: travel further to find the products, substituting a particular product with a similar one, or develop a more intensive or cultivated sources of supply. Ensuring sustainable harvesting of NTFPs is very important for human development in the two districts. This can be achieved by encouraging and supporting their domestication in Ghana. In recent years, as a result of the recognition that the extraction of NTFPs from natural forests has limited potential for improving household incomes and nutrition, several scholars have questioned whether the objective of enhancing forest-based livelihoods could not be better fulfilled by optimizing the production of NTFPs through domestication. The production of these products on a permanent basis will enormously help create more sustainable employment and income-generation opportunities, enhance food security, and improve the nutrition and health of poor farmers and their families. A number of critical factors also continue to constrain the ability of the population to exploit the full potential of NTFPs. These include lack of a clear policy to promote NTFPs, poor processing skills, bushfires, population growth, and lack of organized markets. An appropriate policy framework for the sustainable promotion of NTFPs is also necessary to help ensure effective development, promotion, and sustainable harvesting of these products. Processing and preserving NTFPs in Ghana also remain a major problem confronting the agricultural sector in Ghana. Harvesters of NTFPs are compelled to sell about 80% of their products due to lack of storage and processing facilities. Most products, such as mushrooms, get spoiled within a few days after harvesting. To enhance the use of NTFPs, it is important that processing facilities are provided.

Plant medicines have also remained one of the most affordable and easily-accessible sources of treatment in primary healthcare in Ghana. The results of the present study indicate that a significant proportion of the respondents relied on medicinal plants to treat various ailments. As in most other remote areas in Ghana, due to the shortage of trained medical personnel and facilities, modern health services are not available to most rural areas where the majority of the population lives. Consequently, most people employ several coping mechanisms to overcome their health-related problems, including the use of NTFPs. These plants are the primary source of free or affordable healthcare to a significant proportion of the population in the study area. Unfortunately, most of these plants have not been documented due to lack of effective research and proper documentation and communication. A good number of NTFPs and medicinal plants which are used in nutrition and curing diseases are not documented. It is important that the traditional uses of plants are properly documented, and their therapeutic efficacies are effectively disseminated.

There is also a need for increased collaboration with and within traditional healers and the Centre for Scientific Research into Plant Medicine and NGOs to document useful plant species that are being used by traditional healers but are not yet documented. A vigorous promotion of NTFPs will constitute a very effective mechanism for popularizing the farming of NTFPs as an instrument for nutrition, health, and reduction of poverty. Creation of awareness about the nutritional, medicinal and environmental benefits of NTFPs by the Ministry of Agriculture, the Ministry of Health, and the Ministry of Environment will significantly help promote the farming of NTFPs in Ghana. The potential contribution of NTFPs to nutrition, health, and reduction of poverty has also to be adequately promoted.

### Conclusions

NTFPs continue to make an important contribution to food security and nutrition and health of forest-dependent communities in Ghana. They contribute to improving nutrition either as part of the family diet or as a means to achieve household food security. Their potential for improving food security and sustaining livelihoods is high. The results of the present study revealed that a significant number of households in Sefwi Wiawso and Bibiani-Bekwai districts derive a significant part of their food, nutrition, healthcare needs, and income from NTFPs. These provide essential proteins, vitamins, minerals, and carbohydrates to the households. They also contribute to the well-being of rural households, particularly the poor, in terms of food security, nutrition, and health. However, a number of factors, including a policy vacuum, increased overharvesting, destruction of natural habitats, bushfires, population growth, and high demand, are hindering the use and development of NTFPs in Ghana. An appropriate policy framework for a sustainable promotion of NTFPs, domestication of NTFPs, improving harvesting, and processing techniques are necessary to facilitate food security, reduction of poverty, and improved livelihoods, particularly for the economically-marginalized and forest-dependent communities in Ghana.

## ACKNOWLEDGEMENTS

The authors express their heartfelt gratitude to the Vlaamse Interuniversesitaire Raad (VLIR) for supporting the PhD program of Albert Ahenkan at Vrije Universiteit Brussel (VUB), Belgium. They also sincerely thank Prof. Kwame Ameyaw Domfeh, University of Ghana and Prof. Dr. Luc Hens, Human Ecology Department, Vrije Universiteit Brussel, Belgium, for reviewing the earlier version of the paper. Mr. Eric Lartey of the Forest Service Division, Sefwi Wiawso district and the staff of International Centre for Enterprise and Sustainable Development kindly assisted them during data-collection.
